# Annotations

**Published:** 1904-02-20

**Authors:** 


### ANNOTATIONS.

### Diet in Nocturnal Enuresis.

In an article in the current number of the *British Journal of Children's Diseases* Dr. Percy Lewis argues that enuresis in children is the result of a weak condition of body due to an excessive starchy diet and to an inability to digest the excess of carbohydrate. The urine passed at night is of low specific gravity and neutral or alkaline in reaction, and it is suggested that there is polyuria. Dr. Lewis has observed similar characters in the urine of infants fed on starchy food, and has noted that when the diet is changed the urine becomes normal. Hence he was led to anticipate that a similar explanation might be attached to cases of enuresis, and he has found in practice ample justification for this view. In most cases, he says, a rigid anti-diabetic diet removes the symptom in the course of a few days, and in three to four weeks, sometimes sooner, a normal diet may be given without the risk of a return of the enuresis. At the same time he insists upon the necessity for energetic tonic treatment, medicinal and otherwise. In most cases in private practice it is found that the rigour of the treatment may be safely relaxed so far as the early part of the day is concerned, some starchy food being allowed at breakfast. The medicinal treatment of nocturnal enuresis is only too often quite ineffective unless belladonna is pushed in doses sufficiently large to cause symptoms of poisoning. This is necessarily productive of much inconvenience and is not altogether free from grave risk. Hence Dr. Lewis' suggestion is certain to receive a widespread trial, and it will be interesting to note to what extent it receives confirmation at the hands of other observers.

### Sewage Contamination of Shell-fish.

The report recently issued by the Local Government Board for Ireland upon the conditions, from a sanitary point of view, under which certain forms of edible shell-fish are cultivated on the coasts of Ireland is of considerable importance. The inquiry upon which the report is based was carried out along two distinct lines. Dr. T. J. Browne was instructed to visit the several shell-fish layings on the Irish littoral, and to examine the local surroundings in each place with special reference to the possibilities of sewage contamination, while Professor McWeeney was directed to make bacteriological examinations of samples taken from the various beds. It was arranged that each should pursue his investigations independently and without knowledge of the results arrived at by the other. As might be expected this plan has proved highly profitable. Although as a rule their findings agree pretty closely, there are some notable exceptions. For instance, local inspection showed that certain layings in Carlingford Lough were grossly contaminated with sewage, and it appeared that several cases of typhoid fever occurring in the neighbouring districts could be traced almost certainly to the eating of shell-fish from these localities; yet bacteriological examination failed to show the presence of bacillus coli in most of the samples taken, while in the remainder this organism was found in small numbers only. The conclusion is that the adequate safeguarding of the public, and of the shell-fish industries themselves, cannot be assured by relying merely upon bacteriological tests, whether numerical or absolute, for the presence of the colon bacillus, and that the only efficient means of protection would appear to be the registration and supervision of all sources of supply by some responsible authority

### The Ambidextral Culture Society.

The Ambidextral Culture Society, whose first meeting was held on Tuesday in the rooms of the Medical Society of London, under the presidency of Sir James Sawyer, has been founded with the object of educating public opinion to realise the benefits which would accrue to individuals and to the nation as a whole if children were systematically taught to cultivate the use of their left hands equally with the right. As the hon. secretary, Mr. Jackson, pointed out, it is not a little curious that although one-handedness is for all practical purposes universal among civilised races, yet it is not the natural state nor the most advantageous one to the individual. Most people are ready to admit the evident advantages of ambidexterity, and few are prepared to raise any valid theoretical arguments against the proposal to adopt routine cultivation of ambidextral skill among school children. Those who attempt to do so commonly urge in support of their position the fallacious contention that by using either hand indiscriminately for the performance of skilled movements the individual would only acquire mediocre attainments with either. There have been instances of leading men in various professions requiring manual skill who have been ambidextrous. Sir Edwin Landseer, for example, could not be described as a mediocre draughtsman, yet he could draw equally well with his left hand or his right, and it is said that on a particular occasion, to show what he could do, he sketched a horse's head with one hand and a stag's head with the other. These drawings were executed simultaneously, and when finished it was impossible to say which was the better of the two. It is not difficult to imagine circumstances under which the possession of symmetrically developed powers of brain and body might be of the greatest possible value. Lesions producing right-sided hemiplegia, and possibly aphasia, would lose much of their evil consequences if the right side of the brain were sufficiently cultivated to enable the patient to write and perform skilled actions with his left hand, and to enable him to speak under the direction of a second speech centre in the right hemisphere. Experiments have been carried out on the Continent on a fairly extensive scale with mentally defective children, and it is said that much improvement in their mental condition may often be obtained by teaching them systematically to perform delicate manipulations with the left hand equally with the right. It may be supposed that the improvement obtained is due in this case to the rousing into activity of the hitherto dormant right lobe of the brain, and in this way supplementing the deficiencies of the left hemisphere.

